# Patent Medicines

**Published:** 1894-12

**Authors:** 


### Patent Medicines.

A bill lately introduced into the legislature of Iowa provides that every manufacturer of a patent medicine sold in the State shall have printed on the bottle, package or wrapper, a correct statement of the ingredients in the medicine; and that no seller of drugs shall offer for sale such patent medicine unless the formula of their preparations is printed as described. The act covers manufacturers in Iowa, as well as out By its provision, if a manufacturer fails to comply with it, he cannot sell his medicine in Iowa. The penalty for violation, whether on the part of the retail drug-store keeper or the manufacturer, is punishable by a maximum fine of $100.00 or six months in the penitentiary.—*Sanitarian.*

